# Quality of helping behaviours of members of the public towards a person with a mental illness: a descriptive analysis of data from an Australian national survey

**DOI:** 10.1186/1744-859X-13-2

**Published:** 2014-01-18

**Authors:** Alyssia Rossetto, Anthony F Jorm, Nicola J Reavley

**Affiliations:** 1 Centre for Mental Health, Melbourne School of Population and Global Health, The University of Melbourne, Level 3, 207 Bouverie Street, Carlton, Victoria 3010, Australia

**Keywords:** Mental health literacy, Mental health first aid, Helping behaviours, Intentions

## Abstract

**Background:**

Courses such as Mental Health First Aid equip members of the public to perform appropriate helping behaviours towards people experiencing a mental illness or mental health crisis. However, studies investigating the general public’s knowledge and skills in relation to assisting a person with a mental illness are rare. This study assesses the quality of mental health first aid responses by members of the Australian public using data from a national survey.

**Methods:**

Participants in a national survey of mental health literacy were assigned one of six vignettes (depression, depression with suicidal thoughts, early schizophrenia, chronic schizophrenia, social phobia or post-traumatic stress disorder) and asked an open-ended question about how they would help the character in the vignette. The 6,019 respondents were also asked if and how they had helped a person in real life with a similar problem. Responses to these questions were scored using a system based on an action plan developed from expert consensus guidelines on mental health first aid.

**Results:**

The quality of responses overall was poor, with participants scoring an average of 2 out of 12. The most commonly reported actions for both questions were listening to the person, providing support and information and encouraging them to seek appropriate professional help. Actions such as assessing and assisting with crisis were rarely mentioned, even for the depression with suicidal thoughts vignette.

**Conclusions:**

The quality of the Australian public’s mental health first aid knowledge and skills requires substantial improvement. Particular attention should be given to helping people recognise that anxiety disorders such as social phobia require professional help and to improving responses to a suicidal person.

## Introduction

People with a mental illness are encouraged to seek professional help as soon as possible to improve their long-term outcomes. However, only a minority of people meeting the criteria for mental illness utilise mental health services in any given 12-month period [1]. Evidence indicates that people are very likely to endorse informal sources of help, particularly speaking with close family and friends about their problem, as helpful, sometimes rating them more positively than trained health professionals [2,3]. Studies also suggest that the social networks of people with a mental illness can facilitate help seeking [4] and recovery, through practising helping behaviours [5,6]. Helping behaviours are actions performed by people within the social network of an individual experiencing a mental health problem to provide support, facilitate treatment seeking and manage symptoms. These can be first aid responses, when the potential helper initially becomes aware that the person requires help, or they can be part of an ongoing process of support and care that is located within a broader social context [7]. Given the crucial role that social networks can play in assisting someone with a mental illness, it is important that members of the public understand how to provide effective help in these situations; however, educational courses providing practical advice on this topic are rare.

One exception is the Mental Health First Aid (MHFA) course, which educates people on how to provide help to someone developing a mental health problem or experiencing a mental health crisis until the crisis resolves or appropriate professional help is received [8]. The course teaches participants to address several types of mental illness and mental health crises using the ALGEE action plan [8], a mnemonic representing all the activities a first aider uses when helping. ALGEE stands for

• *A*pproach the person, assess and assist with any crisis

• *L*isten non-judgementally

• *G*ive support and information

• *E*ncourage appropriate professional help

• *E*ncourage other supports

The MHFA course has been extensively evaluated, with the evidence suggesting that participants demonstrate improved knowledge of mental illnesses and mental health first aid skills, more positive attitudes towards appropriate psychological and pharmacological treatments, more confidence in providing support to a person experiencing a mental illness and reduced stigma towards people with mental illness, with these effects lasting up to 6 months after the completion of training [9-12]. Studies also indicate that people utilise the skills they are taught in the course in real life [13]. This suggests that MHFA training has practical value in promoting effective helping behaviours, benefiting individuals and communities.

Formal investigations of the general population which focus on their current knowledge about appropriate helping behaviours in situations involving mental illness are rare. It is important to understand what capabilities people already have in this area so that researchers and educators have an accurate basis from which to further the public’s skills and knowledge and to track improvements or decrements in helping behaviour over time.

One Australian study has investigated helping behaviour towards a hypothetical person with a mental illness in adults. Jorm and colleagues [14] conducted a mental health literacy survey of Australian adults in 2003–2004, assigning participants one of four vignettes and asking an open-ended question about what they would do to help the person in the vignette if they knew and cared about them. Responses were coded into six categories. The study found that encouraging professional help and listening to/supporting the person were the most common answers across all vignettes. Provision of appropriate assistance was more likely from females, people who correctly identified the disorder in the vignette and people with less stigmatising attitudes. This research suggests that while there is some understanding of how to assist someone with a mental illness in the Australian population, further education is necessary.

A similar study of Australian youth has been undertaken more recently using a different method to assess the quality of their first aid responses. Yap and Jorm explored the links between young people’s intentions to help someone experiencing a mental illness and their ensuing first aid actions [15]. Two thousand and five 12- to 25-year-old people were randomly presented with one of four vignettes and asked how they would help the person described. In a follow-up interview 2 years later, participants answered the same question about the same vignette, as well as questions about whether any of their family or friends had experienced a problem like that of the individual in the vignette and what the respondent did to help them. While the previous survey of adults used descriptive methods of analysis, the 526 responses from the youth survey were analysed using a system scoring responses according to their quality based on the MHFA ALGEE action plan. The study noted that responses were generally poorly articulated and not specific enough to warrant high scores using this system. Nevertheless, it was found that first aid intentions predicted behaviour at follow-up, with the exception of encouraging appropriate professional help. This relationship also held for behaviours that were deemed unhelpful by health professionals but which were advocated by young people, such as drinking alcohol to relax or forget the problem [16]. This is the first study to explore the prediction of first aid behaviours from intentions and suggests that using the quality scoring system is a useful and comprehensive way of evaluating levels of mental health first aid skills in the general population.

The current study uses a sample of adults from the 2011 National Survey of Mental Health Literacy and Stigma to quantify Australian adults’ competency in providing assistance to both a hypothetical and a real person experiencing a mental illness. It aims to provide a more comprehensive assessment of the public’s current knowledge standardised against best practice using the same quality scoring system as Yap and Jorm [15]. The analysis includes a correlation between participants’ intentions to provide assistance to the hypothetical person and the helping behaviours performed towards a real person to assess whether knowledge about help giving relates to helping behaviour in real life.

## Methods

A computer-assisted telephone interview (CATI) was conducted with a community sample of 6,019 Australians aged 15 and over by the research company The Social Research Centre. The sample was contacted by random digit dialling of landline and mobile phone numbers between January and April 2011. Using this approach enabled a more representative data sample to be obtained, as landline-only sampling is likely to under-sample young people [17]. The interview lasted approximately 20 min and no remuneration was provided to participants.

### Survey interview

The survey was based on a vignette of a person with a mental illness. After providing demographic information, participants were randomly allocated one of six vignettes: depression, depression with suicidal thoughts, early schizophrenia, chronic schizophrenia, social phobia or post-traumatic stress disorder (PTSD), and randomly presented with either a male (‘John’) or female (‘Jenny’) protagonist. Each vignette conformed to DSM-IV [18] and ICD-10 [19] diagnostic criteria. The male versions of the six vignettes are reproduced in Table 1.

**Table 1:** Vignettes used in 2011 Adult Survey of Mental Health Literacy and Stigma

**Disorder**	**Vignette**
Depression	John is 30 years old. He has been feeling unusually sad and miserable for the last few weeks. Even though he is tired all the time, he has trouble sleeping nearly every night. John doesn’t feel like eating and has lost weight. He can’t keep his mind on his work and puts off making decisions. Even day-to-day tasks seem too much for him. This has come to the attention of his boss, who is concerned about John’s lowered productivity.
Depression with suicidal thoughts	John is 30 years old. He has been feeling unusually sad and miserable for the last few weeks. Even though he is tired all the time, he has trouble sleeping nearly every night. John doesn’t feel like eating and has lost weight. He can’t keep his mind on his work and puts off making any decisions. Even day-to-day tasks seem too much for him. This has come to the attention of John’s boss who is concerned about his lowered productivity. John feels he will never be happy again and believes his family would be better off without him. John has been so desperate, he has been thinking of ways to end his life.
Early schizophrenia	John is 24 and lives at home with his parents. He has had a few temporary jobs since finishing school but is now unemployed. Over the last six months he has stopped seeing his friends and has begun locking himself in his bedroom and refusing to eat with the family or to have a bath. His parents also hear him walking about his bedroom at night while they are in bed. Even though they know he is alone, they have heard him shouting and arguing as if someone else is there. When they try to encourage him to do more things, he whispers that he won’t leave home because he is being spied upon by the neighbour. They realize he is not taking drugs because he never sees anyone or goes anywhere.
Chronic schizophrenia	John is 44 years old. He is living in a boarding house in an industrial area. He has not worked for years. He wears the same clothes in all weathers and has left his hair to grow long and untidy. He is always on his own and is often seen sitting in the park talking to himself. At times he stands and moves his hands as if to communicate to someone in nearby trees. He rarely drinks alcohol. He speaks carefully using uncommon and sometimes made-up words. He is polite but avoids talking with other people. At times he accuses shopkeepers of giving information about him to other people. He has asked his landlord to put extra locks on his door and to remove the television set from his room. He says spies are trying to keep him under observation because he has secret information about international computer systems which control people through television transmitters. His landlord complains that he will not let him clean the room which is increasingly dirty and filled with glass objects. John says he is using these “to receive messages from space”.
Social phobia	John is a 30-year old who lives alone. Since moving to a new town last year he has become even more shy than usual and has made only one friend. He would really like to make more friends but is scared that he’ll do or say something embarrassing when he’s around others. Although John’s work is OK he rarely says a word in meetings and becomes incredibly nervous, trembles, blushes and seems like he might vomit if he has to answer a question or speak in front of his workmates. John is quite talkative with his close relatives, but becomes quiet if anyone he doesn’t know well is present. He never answers the phone and he refuses to attend social gatherings. He knows his fears are unreasonable but he can’t seem to control them and this really upsets him.
Post-traumatic stress disorder	John is a 30-year -old who lives with his wife. Recently his sleep has been disturbed and he has been having vivid nightmares. He has been increasingly irritable, and can’t understand why. He has also been jumpy, on edge and tending to avoid going out, even to see friends. Previously he had been highly sociable. These things started happening around two months ago. John owns a newsagent shop with his wife and has found work difficult since a man armed with a knife attempted to rob the cash register while he was working four months ago. He sees the intruder’s face clearly in his nightmares. He refuses to talk about what happened and his wife says she feels that he is shutting her out.

Survey respondents were next asked to identify what, if anything, they believed to be wrong with John/Jenny, with unprompted responses recorded. This was followed by questions regarding how John/Jenny could best be helped and the likely helpfulness or harmfulness of several interventions (e.g. health professionals, friends and family, self help strategies, medications and therapies), including an open-ended response to the question: ‘Imagine John/Jenny is someone you have known for a long time and care about. You want to help him/her. What would you do?’ Interviewers then asked respondents questions relating to personal and perceived stigma, rating their agreement or disagreement with the statements on a five-point scale. Participants also answered questions about the mental health of the participant’s friends and family, firstly establishing if respondents knew anyone with a problem similar to John’s/Jenny’s. Questions were also asked about the number of affected people, whether the respondent did anything to help the person they knew best, an open-ended question about how they helped the person and whether the close friend or family member sought professional help or treatment. Participants were also asked about their beliefs about the causes of mental illness, their own mental and physical health, and their awareness and knowledge of mental health organisations.

### Coding of open-ended responses

The two open-ended questions relating to how the participant would help the person in the vignette and what the participant did to help their close other with a similar problem to John/Jenny were scored via the scoring system used by Yap and Jorm [15]. The system is based on the ALGEE action plan taught in the second edition of the Mental Health First Aid (MHFA) course [8] and is available on request from the authors. The action plan was developed through a series of studies designed to create mental health first aid guidelines for the public [20-25]. Responses are awarded a point for each component of the action plan they mention (i.e. *Approach the person, Assess and assist with any crisis, Listen non-judgementally, Give support and information, Encourage appropriate professional help* and *Encourage other supports*) and given an additional point per category where specific details are given (e.g. ‘Encourage the person to see a psychologist’ would receive two points for *Encourage appropriate professional help*). Responses can receive a minimum of 0 and a maximum of 2 points per category, giving a total score representing the quality of the response that ranges from 0 to 12.

To ensure the reliability of the assigned scores, a rater first scored 60 sample open-ended responses from a previous trial using the ALGEE scoring system and compared these with consensus scores for the same responses determined by the creators of the MHFA course and the scoring system [9]. This process serves as the gold standard for training raters and assessing scoring validity [9,15]. Inter-rater reliability was calculated using Pearson’s *r* for each category and for the total score, as shown in Table 2. Secondly, the rater and the ALGEE scoring system developers each independently coded 80 randomly selected open-ended responses from the 2011 National Survey of Mental Health Literacy and Stigma. Forty responses were taken from the question ‘Imagine John/Jenny is someone you have known for a long time and care about. You want to help him/her. What would you do?’ and 40 responses were taken from the question ‘What did you do to help the close friend/family member you know who had a problem similar to John’s/Jenny’s?’ Inter-rater reliability is shown in Table 2 and evidences very high inter-rater reliability overall. A rater then scored each of the responses in each question of interest, clarifying with the scoring system developers as necessary.

**Table 2:** Inter-rater reliability for each component of the action plan and total score for sample responses

** ALGEE component**	**Pearson’s *r *** **for sample of 60 responses**	**Pearson’s *r *** **for 40 sample intention responses**	**Pearson’s *r *** **for 40 sample behaviour responses**
Approach the person	0.77	-	-
Assess and assist with any crisis	0.94	1.00	-
Listen non-judgementally	0.81	0.92	0.93
Give support and information	0.68	0.76	0.79
Encourage appropriate professional help	0.86	1.00	0.93
Encourage other supports	0.81	0.88	-
Total score for response	0.89	0.93	0.80

No Pearson’s *r* was obtained for *Approach the person, Assess and assist with any crisis* or *Encourage other supports* as one of the columns used in these correlations had exclusively zero values.

### Statistical analyses

Pre-weights were initially administered to all data to adjust for the respondents’ chances of selection and the dual-frame survey design. A population weight was also applied to account for the survey’s over-sampling of university-educated and English-speaking background participants and under-sampling of males and younger adults. The data were analysed using percent frequencies, means and standard deviations. *Post hoc* nonparametric tests were used to examine differences between vignettes, as the majority of variables were heavily skewed and unable to be transformed due to the categorical nature of the data. Kruskal-Wallis tests were initially employed to assess whether any overall differences between the vignettes existed. Where the overall effect was significant, Mann–Whitney tests were used to compare individual vignettes to all other vignettes to further evaluate the significance and direction of results.

Due to the number of *post hoc* tests run, a Bonferroni correction was applied to the significance level, so that results are reported when *p* < .008. Effect size estimates for the Mann–Whitney tests, in the form of Pearson’s *r* were also calculated as an additional point of comparison between vignettes [26]. Equivalent parametric *post hoc* tests were performed for the total scores for the intention and behaviour questions, as these variables were normally distributed. Violations of Levene’s test for equality of variances were observed in all but one of the significant *post hoc* analyses; *t* test values with unequal variances assumed are reported in these instances. Cohen’s *d* effect sizes are reported for these results. All analyses were performed using SPSS version 20 and Intercooled Stata 12.

### Ethics

Oral consent to participate in the study was obtained from all participants prior to beginning the interview. This study was approved by the University of Melbourne Human Research Ethics Committee.

## Results

The response rate for the survey was 44%, defined as the number of completed interviews (*n* = 6,019) out of the number of potential participants who could be contacted and confirmed as in scope (*n* = 13,636). There were 4,323 interviews conducted on landlines and 1,696 interviews were conducted on mobile phones. There were 2,670 males (44.4%) and 3,349 females (55.6%) sampled. Table 3 shows the number of respondents who received each vignette, the number of people who answered the question ‘Imagine John/Jenny is someone you have known for a long time and care about. You want to help him/her. What would you do?’ (hereafter referred to as ‘intention’ or ‘the intention question’) and the number of people who answered the question ‘What did you do to help the close friend/family member you know who had a problem similar to John’s/Jenny’s?’ (hereafter referred to as ‘behaviour’ or ‘the behaviour question’). There were no significant differences between people receiving the different vignettes with regards to age, gender, marital status, education level, country of birth or location.

**Table 3:** Number of people receiving each vignette and answering the intention and behaviour questions

**Vignette**	**Respondents receiving vignette**	**Respondents answering intention question**	**Respondents answering behaviour question**
Depression	1,016	1,003	561
Depression with suicidal thoughts	1,008	1,001	548
Early schizophrenia	1,002	989	383
Chronic schizophrenia	993	973	286
Social phobia	992	975	438
PTSD	1,008	996	395
Total	6,019	5,937	2,615

### Results for intention question

Of the 6,019 survey respondents, 5,937 (98.6%) answered the intention question and were scored using the ALGEE criteria. Additional file 1: Table S1 contains the percentage frequencies of all the ALGEE components and their scores by vignette for this question. Kruskal-Wallis tests indicated that significant differences existed among all groups across all ALGEE criteria (*p* < .008). Significant results are summarised in Table 4.

**Table 4:** Significant results for differences by vignette for the intention and behaviour questions

	**Vignette**					
**ALGEE criteria**	**Depression**	**Depression with suicidal thoughts**	**Early schizophrenia**	**Chronic schizophrenia**	**Social phobia**	**PTSD**
Approach the person				↓, *r* = .05		
Assess and assist with any crisis		↑, *r* = .10			↓, *r* = .04	
Listen non-judgementally	↑, *r* = .07			↓, *r* = .16	↓, *r* = .06	↑, *r* = .10
				↓, *r* = .07 (b)		↑, *r* = .07 (b)
Give support and information	↓, *r* = .07		↓, *r* = .07	↑, *r* = .06	↑, *r* = .10	↓, *r* = .06 (b)
Encourage appropriate professional help	↑, *r* = .05	↑, *r* = .05			↓, *r* = .13	
Encourage other supports			↑, *r* = .06	↓, *r* = .08	↑, *r* = .07	↓, *r* = .04
Total score	↑, *d* = .10	↑, *d* = .23		↓, *d* = .21	↓, *d* = .20	
				↓, *d* = .16 (b)		

Arrows indicate whether participants receiving the vignette were significantly more (↑) or less (↓) likely to score highly compared to participants receiving all other vignettes at *p* < .008. *r* and *d* indicate effect sizes. (b) indicates that the significance and effect size relates to the behaviour question. All other results relate to the intention question.

#### Approach the person

Across all vignettes, how to approach the person in the vignette was not clearly articulated, with 86.8% of the sample receiving a score of 0 for this criterion and 1.1% receiving a score of 2. The mean score for *Approach the person* was 0.15 (SD = 0.38). *Post hoc* Mann–Whitney tests indicated that respondents who did not receive the chronic schizophrenia vignette were significantly more likely to score highly on this criterion.

#### Assess and assist with any crisis

Almost no respondents detailed assessing and assisting the person in crisis. In the depression with suicidal thoughts vignette, where a crisis is apparent, 19 responses received a score of 2 and 21 answers received a score of 1. This means that 40 of the 1,001 people who answered the intention question for the depression with suicidal thoughts vignette recognised that John/Jenny was experiencing a mental health crisis. However, the number of people mentioning an *Assess and assist with crisis* action for the depression with suicidal thoughts vignette was more than double that of the other vignettes. Respondents receiving the depression with suicidal thoughts vignette were more likely to score highly on this criterion while people receiving the social phobia vignette were less likely to score highly than those receiving other vignettes. Of the total sample of 6,019, 1.3% scored either 1 or 2 points for this criterion, with the mean score being 0.02 (SD = 0.18).

#### Listen non-judgementally

Many respondents recognised and articulated the need for helpers to talk and listen to the person in the vignette. Of the respondents, 38.2% overall received scores of 1 or 2 for this component. Of note here is that, while many respondents stated that they would talk and/or listen to the person, only 140 of these people detailed how they would perform this action (e.g. listen empathically, validate feelings), which was necessary to receive 2 points. Participants receiving the depression and PTSD vignettes scored significantly higher, while people assigned the chronic schizophrenia and social phobia vignettes scored significantly lower compared to the other vignettes. The overall mean score for the *Listen non-judgementally* criterion was 0.40 (SD = 0.54).

#### Give support and information

Almost half of the responses to the intention question (47.9%) received scores of 1 or 2 for this criterion. Again, the majority of people provided superficial responses (*M =* 0.50, SD = 0.56), suggesting that while the public recognises the need to support people with mental illness, they may not necessarily understand what types of support or information can be provided. In the chronic schizophrenia and social phobia vignettes, more people than not stated this as a helping action. *Post hoc* comparisons confirmed that the people receiving these vignettes were significantly more likely to score highly on this component compared to participants receiving other vignettes. Compared to the other vignettes, participants receiving the depression and early schizophrenia vignettes were significantly less likely to provide answers of a high quality.

#### Encourage appropriate professional help

Approximately half of the respondents (48.9%) overall supplied an answer that was awarded 1 or 2 points for this component, with a mean score of 0.91 (SD = 0.93) across all responses. Of these responses, 2,331 were allocated 2 points for correctly stating a specific type of professional help, such as a GP, psychologist or psychiatrist. In all but the social phobia vignette, well over half of the responses mentioned encouraging professional help of some description for the person in the vignette. Mann–Whitney tests indicated that people receiving the depression and depression with suicidal thoughts vignettes were significantly more likely to score highly, while respondents to the social phobia vignette were significantly less likely to recommend professional help of any description.

#### Encourage other supports

The majority of respondents overall (89.3%) scored 0 for this criterion, with only eight people in total receiving a score of 2. Four of the eight responses came from the depression with suicidal thoughts vignette. The mean score for *Encourage other supports* was 0.11 (SD = 0.32). Mann–Whitney tests indicated that significantly higher scores were achieved by respondents assigned the early schizophrenia and social phobia vignettes, while people receiving the chronic schizophrenia and PTSD vignettes scored significantly lower.

#### Total score

The scores for each ALGEE category were summed for each response to give a total score. These scores ranged between 0 and 7 out of a possible maximum score of 12. The majority of people scored between 1 and 3 (*M =* 2.08, SD = 1.16). Seven people were awarded a score of 7 (0.1% of the total sample), with four of these coming from people receiving the depression with suicidal thoughts vignette, two from the depression vignette and one from the PTSD vignette. A one-way ANOVA suggested that significant differences between vignettes existed (*F*(5, 5931) = 21.09, *p* < .008). Participants receiving the depression and depression with suicidal thoughts vignettes scored significantly higher than participants receiving other vignettes, while people who received the chronic schizophrenia and social phobia vignettes were significantly less likely to receive high scores.

### Results for behaviour question

Respondents were asked if they knew anyone with a problem similar to John’s/Jenny’s. Fifty-four percent of the total sample (3248 respondents) said they did. There were 2,649 people who stated that they had done something to help the person, and 2,615 respondents (43.4% of the total sample) provided an answer to the question ‘What, if anything, did you do to help the person?’ Additional file 1: Table S2 contains the percentage frequencies of all the ALGEE components and their scores by vignette for this question. Kruskal-Wallis tests indicated that significant differences existed among the *Listen non-judgementally* and *Give support and information* criteria (*p* < .008). Significant results are summarised in Table 4 and indicated with a (b) symbol.

#### Approach the person

This component was poorly articulated, with 91.7% of respondents receiving a score of 0. Nine respondents were awarded a score of 2, representing 0.4% of respondents who answered the question; four of these people received the depression vignette. The mean score for this criterion was 0.09 (SD = 0.29).

#### Assess and assist with any crisis

Again, the majority of answers did not mention this component (*M =* 0.02, SD = 0.19). Overall, 25 respondents scored 1 point (1.1%) and 17 respondents scored 2 points on this criterion (0.5%). Seven of the responses awarded a score of 2 received the depression with suicidal thoughts vignette, but people receiving this vignette were not more likely than respondents receiving other vignettes to achieve higher scores on this criterion.

#### Listen non-judgementally

Across all vignettes, 37.4% of people scored either 1 or 2 points for *Listen non-judgementally*, with 55 people in total receiving a score of 2. The mean score for this component was 0.39 (SD = 0.53). This reflects similar numbers to the *Listen non-judgementally* criterion for the intention question. Respondents receiving the PTSD vignette scored significantly higher on this criterion and people assigned the chronic schizophrenia vignette were less likely to score highly.

#### Give support and information

The majority of answers (66.6%) stated that at least one form of support or information had been provided to the person known in real life. Across all vignettes, the most common score was 1, with the mean score being 0.75 (SD = 0.61). People receiving the PTSD vignette were significantly less likely to provide high-quality support and information compared to those receiving other vignettes.

#### Encourage appropriate professional help

The majority of respondents for the behaviour question received scores of 0 for this criterion (57.4%). Of the people who received scores of 1 or 2, 891 could correctly name an appropriate source of professional help, giving them a score of 2. The mean score for *Encourage appropriate professional help* was 0.79 (SD = 0.92).

#### Encourage other supports

Again, most respondents (89.3%) scored 0 for this criterion, with eight people across all vignettes listing at least two types of other supports they provided or suggested to the person needing help. Five of these scores came from people receiving the social phobia vignette. The mean score for this criterion was 0.11 (SD = 0.33).

#### Total score

The total scores for the behaviour question also ranged between 0 and 7 across all vignettes. The mean score was 2.15 (SD = 1.15), with 85.4% of scores falling between 1 and 3. Three people received scores of 7 (0.1%); 21 people scored a total of 6 (0.8%). A *post hoc* ANOVA indicated that there were significant differences between vignettes (*F*(5, 2,609) = 2.43, *p* = .03). Respondents who received vignettes other than the chronic schizophrenia vignette attained significantly higher totals.

### Correlations between intention and behaviour

The correlation between the intention and behaviour variables was computed using the total scores from each question. The correlation between these variables was 0.203 (*p* < .01), indicating that intention and behaviour had a significant but low correlation overall. Examination of this correlation as a function of each vignette showed similarly low, significant correlations for depression (0.289, *p* < .01), depression with suicidal thoughts (0.239, *p* < .01), early schizophrenia (0.162, *p* < .01), social phobia (0.221, *p* < .01) and PTSD (0.151, *p* < .01), with chronic schizophrenia the only non-significant correlation (0.078, *p* > .05). Correlations between the intention and behaviour questions by ALGEE component were all significant at the *p* < .01 level, with correlations ranging between 0.061 (*Listen non-judgementally*) and 0.231 (*Encourage appropriate professional help*).

## Discussion

This study aimed to better understand the ability of the Australian population to provide assistance to a hypothetical and a real person with a mental illness and to compare this with current best practice in the field of mental health first aid. The study also investigated the links between what people stated they would do to help someone with a mental illness and how this was reflected in their real-life actions.

### Findings from this study

The findings indicate that, overall, responses from the public outlining appropriate helping behaviours towards people with a mental illness score poorly according to the ALGEE system. An average score of 2 indicates that people can state two actions (for example, listening and providing support) or can comprehensively report on one action (such as naming a specific health professional to visit), but otherwise lack the particular knowledge and skills to effectively assist someone with a mental illness. This implies that courses like MHFA can be beneficial in terms of educating the public on simple, beneficial behaviours that will help people with mental illness feel supported, accepted and motivated to seek professional assistance.

The most commonly reported action for the intention question was *Encourage appropriate professional help*, being mentioned in 48.9% of responses. This is a positive outcome, as it suggests that the public is aware that mental illness is best treated with the assistance of health professionals. Interestingly, this trend was not reflected in responses to the social phobia vignette, with only 33.6% of people reporting that they would encourage the person to seek professional assistance. This may be because this disorder was poorly recognised and named as a mental illness by respondents [2], prompting answers primarily focused on providing social support or encouraging social activities. *Encourage appropriate professional help* was also not the most commonly reported action for the behaviour question (this was *Give support and information*, with over 66% of people providing this response). This reflects similar findings in Yap and Jorm’s youth study [15], suggesting that this is a widespread trend in the Australian population and that behaviours that encourage professional help seeking need to be more actively promoted to the public as a complement to providing support and information.

In contrast, responses classified in the *Assess and assist with any crisis* category were very infrequently reported across the sample for both the intention and behaviour question. This is especially concerning for the depression with suicidal thoughts vignette, where a life threatening crisis is evident. Compared to mental health professionals, the public are less likely to believe in the helpfulness of asking a person about suicidal thoughts and feelings, and more likely to believe this action is harmful [27], despite evidence suggesting that there are no detrimental effects associated with screening for suicidal intent [28]. Assessing for suicidal intent is an action that requires greater destigmatisation and promotion in the community as a helpful response.

Another notable finding is that people receiving the chronic schizophrenia vignette were significantly more likely to score poorly in several areas, including *Approach the person*, *Listen non-judgementally* and *Encourage other supports*, culminating in significantly lower total scores for both intention and behaviour. This may reflect a greater prevalence of stigmatising attitudes towards people with psychotic disorders compared to people with other mental illnesses within the community, particularly the idea that they are unpredictable and dangerous [29]. This could result in a reduced likelihood of even attempting mental health first aid and suggests that encouraging the community to perceive people with schizophrenia as no different to people with any other mental illness might enhance the quality of mental health first aid responses for this group.

In comparing the responses for the intention and behaviour questions, there were few differences. The most commonly reported actions for both questions were *Listen non-judgementally, Give support and information* and *Encourage appropriate professional help*. Mean scores for each ALGEE component were similar when comparing responses to each question (for example, the mean score for *Encourage other supports* was 0.11 for both the intention and behaviour questions), indicating that, in general, the public seem likely to perform the same actions towards both a hypothetical and a real person. This is further supported by the correlation between the total scores for the intention and behaviour questions, which was low but significant. This is encouraging, as it implies that if members of the public are equipped with the knowledge and skills to adequately assist someone with a mental illness, they are likely to actually perform these actions when necessary. This notion is also supported by well-established psychological theories, such as the Theory of Planned Behaviour [30] which postulates that intentions are the precursor to, and a reasonably accurate predictor of, an individual’s actions in a given situation.

### Comparison with previous studies

As previously mentioned, two similar studies to this one have been published. Yap and Jorm [15] analysed the first aid intentions and subsequent behaviours of a nationally representative sample of youth using the same scoring system as this investigation. The results of the present study support the general patterns found in the earlier study, in that mean scores and standard deviations were similar for both intention and behaviour (with the means in this study generally higher), the *Listen non-judgementally, Give support and information* and *Encourage appropriate professional help* components were more frequently reported than the other ALGEE criteria, the *Approach the person* and *Assess and assist with any crisis* criteria were rarely mentioned and total scores were quite low overall. This suggests that Australian adults’ knowledge and behaviour are very similar to Australian youth in relation to helping a person with a mental illness.

Jorm and colleagues’ study [14] also used a representative sample of Australian adults, coding their responses using descriptive categories rather than scoring the quality of the responses. Although it is not possible to statistically compare the results of this study and the 2005 investigation due to differences in how the data was collected (phone interview versus face-to-face interview, which yields more comprehensive responses) and analysed (descriptive analysis versus scoring the quality of responses), it is possible to ascertain general data patterns and changes over time. Again, the findings of the two studies are similar, with the *Listen non-judgementally* and *Encourage appropriate professional help* responses being most commonly stated and respondents able to correctly nominate an appropriate health professional to assist the person in the vignette. *Assess and assist with any crisis* responses were seldom reported; however, the percentage frequencies for this category were substantially higher than those for *Give support and information* in three of the four vignettes used in the 2005 study. This could be an artefact of the face-to-face interview methodology, where participants tended to give longer responses. Overall, intentions to help the person in the vignette seem to have remained relatively unchanged from 2003–2004 to 2011.

From a broader perspective, the findings of this study lend support to the postulates of social network frameworks such as the Network Episode Model (NEM) [4], which suggest that interactions between individuals and social systems, particularly family and friends, reciprocally influence and shape a person’s pathway to health care. Studies in this field reinforce the important role of social networks in recognising, defining and legitimising mental illness [31] and assisting entry into the mental health care system [32]. Findings from both the mental health literacy and NEM literatures strongly indicate that improvements can be made in how effectively and efficiently the public initially responds to mental health problems in people they encounter.

### Strengths and limitations of this study

This investigation exhibits several strengths. The National Survey of Mental Health Literacy and Stigma had a large, nationally representative sample, resulting in 5,937 responses to the intention question and 2,615 responses to the behaviour question that could be scored using the ALGEE criteria. The variety of vignettes presented to participants means that responses to the intention question could be compared both within and across a standard set of situations, and this is one of the first studies to examine first aid responses towards people with anxiety disorders. Using a standard scoring system was helpful in ensuring reliability of measurement, as reflected in the high inter-rater reliabilities for each set of responses. Lastly, using the ALGEE scoring system enabled a score to be assigned based on the quality of a person’s response and provided a clearer understanding of how the general public’s knowledge and skills compared with best practice according to expert consensus guidelines.

This study also has limitations, some of which can be addressed in future research. Firstly, the link between intention and behaviour is unclear. This is partly due the study’s methodology, which involved retrospective reporting of behaviour. To answer the behaviour question, any helping actions must necessarily have taken place prior to participating in the survey, thus behaviour actually preceded intention. The behaviour the participant reported on could have been recently performed or have taken place many years ago. Also, this survey only contains data from a single time point, which does not account for what experiences shaped participants’ responses when answering these questions. To better understand this relationship, a prospective study, similar to that conducted by Yap and Jorm [15], should be undertaken with an adult sample. Additionally, given that the correlation between these variables is only 0.203, other factors must help to determine whether and what help people provide to a person experiencing mental illness. Supporting this notion is a previously conducted study of predictors of mental health first aid actions in young people suggesting that several characteristics of both the respondent and recipient of aid affect the likelihood and type of helping response provided [33]. Secondly, answers to the behaviour question were not standardised according to a specific scenario and were potentially subject to respondent recall bias; thus, it is difficult to judge how appropriate these responses were to the actual situation and also how closely the person’s symptoms matched those presented in the vignette. The nature and severity of the person’s problem in real life was unclear, and hence, the appropriateness of the first aid actions cannot be determined. Additionally, it is unknown whether the respondent’s actions benefited or harmed the real-life recipient. Future studies could incorporate questions relating to the wellbeing of the person assisted, or attempt to directly question that person to establish the effectiveness of the behaviours performed. Lastly, while the ALGEE scoring system displayed several useful features, there were some aspects of this data that it was unable to capture effectively. For example, a substantial proportion of people stated that while they were currently unsure of how to help the person in the vignette, they would seek advice on what to do from other sources, such as a GP, the internet or someone they knew who had experienced a similar problem. While this is an appropriate response from a person who is uncertain about how to provide effective help, the scoring system cannot adequately categorise and allocate points to it, as it assumes that respondents will take direct action themselves. Modifications to the ALGEE scoring system that incorporate such responses may be warranted.

## Conclusions

The results of this study support conclusions drawn from previous research into how members of the public assist a person experiencing a mental illness. Taken together, this body of research indicates that the public’s mental health first aid knowledge and skills can be substantially improved. One way to achieve this is through the promotion of programs, like MHFA, which aim to improve the public’s ability to recognise and address emerging mental health issues in people they know. Given that research consistently demonstrates a significant correlation between people’s intention and behaviour [34], and that the MHFA course is increasing in both visibility and popularity [35], the potential exists for educating many more people about how to appropriately assist someone with a mental illness and subsequently having these competencies communicated to researchers in future studies and demonstrated in real life.

## Competing interests

The authors declare that they have no competing interests.

## Authors’ contributions

AR wrote the manuscript, coded all the responses and performed the analyses. AFJ helped secure funding for the survey, co-designed the survey, co-developed the coding scheme, co-developed the scoring for the sample responses for inter-rater reliability and approved this manuscript. NJR helped secure funding for the survey, co-designed the survey and approved this manuscript. All authors read and approved the final manuscript.

## Supplementary Material

Additional file 1: Table S1 Percentage frequencies for intention question by ALGEE component and vignette. **Table S2.** Percentage frequencies for behaviour question by ALGEE component and vignette. Click here for file
