# Supplementary material for: Quality of helping behaviours of members of the public towards a person with a mental illness: a descriptive analysis of data from an Australian national survey
Source: Ann Gen Psychiatry. 2014 Jan 18;13:2. doi: 10.1186/1744-859X-13-2 (PMC3898824; doi:10.1186/1744-859X-13-2)
Supplement: Additional file 1: Table S1 — Percentage frequencies for intention question by ALGEE component and vignette. Table S2. Percentage frequencies for behaviour question by ALGEE component and vignette. [file 1744-859X-13-2-S1.docx]

Original paper

**Title**

Quality of helping behaviours of members of the public towards a person with a mental illness: A descriptive analysis of data from an Australian national survey.

Alyssia Rossetto, BSc ; Anthony F. Jorm, PhD, DSc; Nicola J. Reavley, PhD; Population Mental Health Group, Melbourne School of Population and Global Health, The University of Melbourne, Victoria, Australia.

Email: Alyssia Rossetto - [alyssia.rossetto@unimelb.edu.au](mailto:alyssia.rossetto@unimelb.edu.au); Anthony F. Jorm - [ajorm@unimelb.edu.au](mailto:ajorm@unimelb.edu.au); Nicola Reavley - [nreavley@unimelb.edu.au](mailto:nreavley@unimelb.edu.au)

Correspondence: Alyssia Rossetto, Population Mental Health Group, Melbourne School of Population and Global Health, Level 3, 207 Bouverie Street, VIC 3010

The University of Melbourne, Australia, Phone: +61 3 8344 9353; Fax: +61 3 9349 5815; Email: [alyssia.rossetto@unimelb.edu.au](mailto:alyssia.rossetto@unimelb.edu.au)

Table S1. Percentage frequencies for intention question by ALGEE component and vignette.

|  |  |  |  | **Vignette** |  |  |  |  |
| --- | --- | --- | --- | --- | --- | --- | --- | --- |
| **ALGEE criteria** | Score | Depression | Depression with suicidal thoughts | Early schizophrenia | Chronic schizophrenia | Social phobia | Post-traumatic stress disorder | Total for all vignettes |
| Approach the person | 0 | 83.9 | 87.7 | 86.7 | 89.6 | 87.8 | 85.0 | 86.8 |
|  | 1 | 14.7 | 10.9 | 11.5 | 9.8 | 12.0 | 13.6 | 12.1 |
|  | 2 | 1.4 | 1.4 | 1.8 | 0.5 | 0.2 | 1.4 | 1.1 |
| Assess and assist with any crisis | 0 | 98.9 | 96.5 | 99.2 | 98.9 | 99.6 | 99.5 | 98.8 |
|  | 1 | 0.6 | 2.1 | 0.3 | 0.9 | 0.4 | 0.4 | 0.8 |
|  | 2 | 0.4 | 1.4 | 0.5 | 0.3 | 0.0 | 0.1 | 0.5 |
| Listen non-judgementally | 0 | 54.9 | 58.0 | 59.6 | 80.1 | 67.9 | 51.5 | 61.8 |
|  | 1 | 42.5 | 39.5 | 37.5 | 17.9 | 30.4 | 46.6 | 35.9 |
|  | 2 | 2.7 | 2.5 | 3.0 | 2.0 | 1.6 | 1.8 | 2.3 |
| Give support and information | 0 | 60.6 | 49.6 | 59.3 | 46.2 | 40.3 | 56.5 | 52.1 |
|  | 1 | 36.1 | 46.4 | 38.1 | 49.9 | 57.7 | 41.2 | 44.9 |
|  | 2 | 3.4 | 3.9 | 2.6 | 3.9 | 2.0 | 2.3 | 3.0 |
| Encourage appropriate professional help | 0 | 45.8 | 46.6 | 48.3 | 48.6 | 66.4 | 50.6 | 51.1 |
|  | 1 | 10.1 | 12.0 | 14.7 | 11.1 | 9.5 | 14.3 | 12.0 |
|  | 2 | 44.1 | 41.4 | 37.0 | 40.3 | 24.1 | 35.1 | 36.9 |
| Encourage other supports | 0 | 91.1 | 87.3 | 85.7 | 95.3 | 85.1 | 91.9 | 89.3 |
|  | 1 | 8.8 | 12.5 | 14.2 | 4.7 | 14.8 | 8.1 | 10.6 |
|  | 2 | 0.1 | 0.2 | 0.1 | 0.0 | 0.1 | 0.0 | 0.1 |
| Total score | 0 | 5.9 | 4.8 | 8.5 | 15.8 | 4.3 | 5.2 | 7.3 |
|  | 1 | 24.4 | 23.0 | 25.9 | 22.5 | 41.1 | 27.3 | 27.5 |
|  | 2 | 33.1 | 33.9 | 32.3 | 27.6 | 32.9 | 36.2 | 32.7 |
|  | 3 | 24.8 | 25.5 | 22.6 | 27.8 | 14.8 | 21.3 | 22.7 |
|  | 4 | 8.8 | 8.1 | 7.4 | 4.9 | 5.6 | 8.6 | 7.3 |
|  | 5 | 1.6 | 3.6 | 2.8 | 1.4 | 1.1 | 1.3 | 2.0 |
|  | 6 | 1.2 | 0.9 | 0.5 | 0.0 | 0.2 | 0.0 | 0.5 |
|  | 7 | 0.2 | 0.2 | 0.0 | 0.0 | 0.0 | 0.1 | 0.1 |

Table S2. Percentage frequencies for behaviour question by ALGEE component and vignette.

|  |  |  |  | **Vignette** |  |  |  |  |
| --- | --- | --- | --- | --- | --- | --- | --- | --- |
| **ALGEE criteria** | Score | Depression | Depression with suicidal thoughts | Early schizophrenia | Chronic schizophrenia | Social phobia | Post-traumatic stress disorder | Total for all vignettes |
| Approach the person | 0 | 93.6 | 89.0 | 90.0 | 90.9 | 92.3 | 94.4 | 91.7 |
|  | 1 | 5.7 | 10.6 | 9.4 | 9.1 | 7.2 | 5.6 | 7.9 |
|  | 2 | 0.7 | 0.4 | 0.6 | 0.0 | 0.5 | 0.0 | 0.4 |
| Assess and assist with any crisis | 0 | 99.0 | 97.9 | 97.1 | 98.2 | 99.2 | 99.1 | 98.4 |
|  | 1 | 0.7 | 1.3 | 2.4 | 1.0 | 0.6 | 0.8 | 1.1 |
|  | 2 | 0.3 | 0.8 | 0.5 | 0.8 | 0.2 | 0.1 | 0.5 |
| Listen non-judgementally | 0 | 56.8 | 62.3 | 64.3 | 73.6 | 67.7 | 55.6 | 62.5 |
|  | 1 | 41.9 | 35.0 | 33.5 | 23.4 | 28.9 | 42.2 | 35.0 |
|  | 2 | 1.3 | 2.7 | 2.2 | 3.0 | 3.4 | 2.2 | 2.4 |
| Give support and information | 0 | 31.4 | 34.7 | 37.7 | 28.4 | 28.1 | 39.0 | 33.3 |
|  | 1 | 62.6 | 55.9 | 53.4 | 62.2 | 61.3 | 51.6 | 57.8 |
|  | 2 | 6.0 | 9.4 | 8.8 | 9.4 | 10.5 | 9.3 | 8.8 |
| Encourage appropriate professional help | 0 | 57.6 | 55.4 | 62.3 | 57.6 | 55.7 | 56.8 | 57.4 |
|  | 1 | 8.8 | 9.3 | 10.9 | 6.1 | 12.1 | 12.7 | 10.1 |
|  | 2 | 33.6 | 35.3 | 26.8 | 36.3 | 32.2 | 30.4 | 32.5 |
| Encourage other supports | 0 | 90.7 | 90.4 | 89.3 | 88.6 | 88.1 | 88.1 | 89.3 |
|  | 1 | 9.3 | 9.4 | 10.3 | 11.3 | 10.9 | 11.9 | 10.4 |
|  | 2 | 0.0 | 0.2 | 0.3 | 0.1 | 1.0 | 0.0 | 0.3 |
| Total score | 0 | 2.0 | 2.6 | 5.4 | 4.7 | 3.7 | 3.6 | 3.4 |
|  | 1 | 30.2 | 28.1 | 30.8 | 26.7 | 28.7 | 29.7 | 29.1 |
|  | 2 | 35.8 | 34.3 | 35.5 | 30.0 | 35.0 | 34.8 | 34.5 |
|  | 3 | 21.3 | 22.1 | 20.1 | 30.6 | 18.9 | 21.2 | 21.8 |
|  | 4 | 7.8 | 9.7 | 5.7 | 6.3 | 8.1 | 8.2 | 7.8 |
|  | 5 | 1.7 | 2.0 | 2.2 | 1.7 | 4.7 | 2.0 | 2.4 |
|  | 6 | 0.9 | 1.2 | 0.4 | 0.0 | 0.9 | 0.6 | 0.8 |
|  | 7 | 0.3 | 0.0 | 0.0 | 0.1 | 0.0 | 0.0 | 0.1 |
